# Data supporting the forecast of electricity generation capacity from non-conventional renewable energy sources in Colombia

**DOI:** 10.1016/j.dib.2019.104949

**Published:** 2019-12-05

**Authors:** Alexis Sagastume Gutiérrez, Milen Balbis Morejón, Juan José Cabello Eras, Mario Cabello Ulloa, Francisco Javier Rey Martínez, Juan Gabriel Rueda-Bayona

**Affiliations:** a Department of Energy, Universidad de la Costa, Calle 58 No. 55-66, Barranquilla, Colombia; b IK4-IKERLAN Technology Research Centre, P J Ma Arizmendiarrieta, 2, 20500 Arresate-Mondragón, Gipuzkoa, Spain; c University of Valladolid, School of Engineering, Department of Energy and Fluidmechanics, Paseo del Cauce n. 59, 47011 Valladolid, Spain; d Universidad Militar Nueva Granada, Engineering Faculty, Civil Engineering, Water and Energy (AyE) Research Group, Bogotá: Carrera 11 No.101- 80, Colombia

**Keywords:** Renewable energy, Non-conventional renewable energy, Renewable-based electricity, Power generation

## Abstract

The data included in this study was calculated based on data provided by the national project registry provided by the Colombian government. The data forecasts the evolution of the power generation capacity registered in non-conventional renewable energy source projects in three scenarios of implementation of the power generation capacity registered in the projects. Results can be used to benchmark non-conventional renewable energy sources in Colombia, interpret the effectiveness of renewable policies, and monitor the evolution of non-conventional renewable-based power generation. The data presented in the article relates to the research study: A look to the electricity generation from non-conventional renewable energy sources in Colombia [1].

**Table:** Specifications Table

Subject	Renewable Energy, Sustainability and the Environment
Specific subject area	Renewable energy encompasses different technologies to exploit renewable energy from sources like the wind, the sun or the biomass. Renewable energy technologies are the mainstream to diversify the energy mix worldwide towards reduced use of fossil fuels. In Colombia, non-conventional renewable energy sources include any renewable energy technology different from large hydroelectric power plants, which are widespread and support around 70% of the electricity produced in the country. Therefore, non-conventional sources include technologies to exploit the energy of the wind, the sun, the biomass, and small hydropower.
Type of data	Table
How data were acquired	In Colombia, renewable energy projects must be approved by the government. Thus, there is a database for non-conventional renewable energy projects. The data available from the database was used to forecast the electricity generation capacity from non-conventional renewable energy sources in Colombia, which is the data presented in this data paper.
Data format	Raw Analyzed Filtered
Parameters for data collection	The primary data were mined from the national database of non-conventional renewable energy source projects of the Colombian Ministry of Energy and Mines. The database includes detailed and updated information on non-conventional renewable energy projects and their transit through the approval process steps. The condition to include projects in the research is the initial approval by the ministry, which certificate the technical feasibility of the project.
Description of data collection	The primary data of the projects registered in the database is organized according to its generation capacity and their approval stage status. This information is used to forecast the most likely starting date of each project in three different scenarios of the success of the projects.
Data source location	Colombia
Data accessibility	With the article
Related research article	Author's name: Juan José Cabello Eras, Milen Balbis Morejón, Alexis Sagastume Gutiérrez, Aldo Pardo García, Mario Cabello Ulloa, Francisco Javier Rey Martínez, Juan Gabriel Rueda-Bayona Title: A look to the electricity generation from non-conventional renewable energy sources in Colombia Journal: International Journal of Energy Economics and Policy https://doi.org/10.32479/ijeep.7108

**Table:** 

**Value of the Data** • It can be used to benchmark the integration path of non-conventional renewable energy sources in Colombia. • It can be used to assess the effectiveness of the policies implemented to develop non-conventional renewable energy sources in Colombia. • It can be used to monitor the balance in the integration of different non-conventional renewable energy sources within the Colombian energy matrix. • It can be used as a starting point to forecast the renewable energy availability in Colombia through the year, considering that generally, the low availability of conventional renewable energy sources coincides with the high availability of non-conventional sources and vice versa.

## 1 Data

The data presented in the paper, shows the performance of non-conventional renewable energy (NCRE) projects in Colombia, which includes photovoltaic (PV), Eolic, biomass and small hydroelectric (SHC) renewable sources [1]. The registration of NCRE projects started in 2016, thus a period between 2016 and 2018 is assessed. Based on this data three scenarios forecasting the performance of NCRE based power generation between 2019 and 2023 are developed. Primary data (i.e. the NCRE projects registered between 2016 and 2018) was obtained from the national NCRE projects registry database, which is available from Ref. [2]. The database includes the transit of registered projects through the stages of approval.

Table 1 shows the NCRE projects registered between 2016 and 2018, including their approval stage. It also includes the sum of the power generation capacity (PGC) considered in the registered projects for each approval stage. Moreover, Table 2 shows the PV projects registered or forecasted between 2016 and 2023 in 6 ranges of PGC. Table 3 shows the forecast of PGC integrated into the electric system between 2019 and 2023 for PV projects in three scenarios. Table 4 shows the wind projects registered or forecasted between 2016 and 2023 in four ranges of PGC. Table 5 shows the forecasted PGC integrated into the electric system between 2019 and 2023 for Eolic projects in three scenarios. Table 6 shows the biomass projects registered or forecasted between 2016 and 2023 in five power ranges. Table 7 shows the forecast of PGC integrated into the electric system between 2019 and 2023 for biomass projects in three scenarios. Table 8 shows the SHC projects registered or forecasted between 2016 and 2018 in three power ranges. Table 9 shows the forecasted PGC integrated into the electric system between 2019 and 2023 for SHP projects in three scenarios. Finally, Table 10 summarizes the forecast of PGC integrated into the electric system between 2019 and 2023 for the three scenarios considered.

**Table 1:** Approval stage and power generation capacity (PGC) for projects registered between 2016 and 2018.

Energy source	Project approval stage	2016		2017		2018		2019	
		Projects	PGC (MW)	Projects	PGC (MW)	Projects	PGC (MW)	Projects	PGC (MW)
Solar	1	92	481.9	153	1213.8	162	1435.2	434.0	3325.9
	2	23	428.4	37	700.3	36	582.6	214.0	10331.0
	3	34	5.2	28	433.0	13	1133.3	74.0	72.3
Wind	1	5	353.9	2	441.8	2	492.9	12.0	1649.2
	2	2	39.8	1	19.9	7	212.1	26.0	3038.8
	3	0	19.9	0	39.8	0	59.7	0.0	0.0
Biomass	1	8	37.6	4	42.2	6	63.4	13.0	230.4
	2	0	20.6	3	65.8	3	20.3	2.0	44.0
	3	3	0.0	3	41.6	0	107.4	3.0	61.4
SHC	1	31	110.5	35	613.8	60	945.4	263.0	2602.1
	2	13	260.7	26	109.4	17	294.6	77.0	865.5
	3	4	15.1	2	264.2	2	373.6	23.0	212.9
**Total**	**1**	**136**	**983.9**	**194**	**2311.7**	**230**	**2937.0**	**722.0**	**7807.5**
	**2**	**38**	**749.5**	**67**	**895.4**	**63**	**1109.6**	**319.0**	**14279.3**
	**3**	**41**	**40.2**	**33**	**778.6**	**15**	**1674.0**	**100.0**	**346.6**

**Table 2:** Statistics and forecasts of the power generation capacity registered in PV projects.

Power range (MW)	Approval process stage	PGC registered in projects (MW)				PGC forecasted in future projects (MW)			
		2016	2017	2018	2019	2020	2021	2022	2023
0–1	1	6.8	12.8	18.0	11.0	19.5	28.0	36.4	44.9
	2	0.2	3.3	6.2	2.8	11.0	19.5	28.0	36.4
	3	3.3	4.8	8.0	0.0	2.8	11.0	19.5	28.0
1–10	1	24.9	71.4	111.4	97.8	151.0	204.1	257.3	310.5
	2	0.0	44.3	34.3	283.4	97.8	151.0	204.1	257.3
	3	2.0	0.0	44.3	6.9	283.4	97.8	151.0	204.1
10–20	1	398.0	211.2	295.7	157.1	341.9	526.7	711.6	896.4
	2	358.2	156.6	101.4	1070.7	157.1	341.9	526.7	711.6
	3	0.0	358.2	514.8	39.8	1070.7	157.1	341.9	526.7
20–50	1	0.0	58.0	63.8	233.0	294.7	356.5	418.2	480.0
	2	0.0	0.0	27.8	238.0	233.0	294.7	356.5	418.2
	3	0.0	0.0	0.0	0.0	238.0	233.0	294.7	356.5
50–100	1	52.2	55.0	60.5	449.9	557.4	664.9	772.3	879.8
	2	70.0	100.0	26.4	1668.0	449.9	557.4	664.9	772.3
	3	0.0	70.0	170.0	0.0	1668.0	449.9	557.4	664.9
>100	1	0.0	805.4	885.9	950.0	1409.7	1869.3	2329.0	2788.6
	2	0.0	396.2	386.6	4312.7	950.0	1409.7	1869.3	2329.0
	3	0.0	0.0	396.2	0.0	4312.7	950.0	1409.7	1869.3
Total	**1**	**481.9**	**1213.8**	**1435.2**	**1898.8**	**2774.2**	**3649.5**	**4524.8**	**5400.2**
	**2**	**428.4**	**700.3**	**582.6**	**7575.5**	**1898.8**	**2774.2**	**3649.5**	**4524.8**
	**3**	**5.2**	**433.0**	**1133.3**	**46.7**	**7575.5**	**1898.8**	**2774.2**	**3649.5**

**Table 3:** Scenarios of power generation capacities yearly integrated into the electric system. PV projects.

Power range (MW)	Scenario	Forecasted implementation of PGC (MW)			
		2020	2021	2022	2023
0–1	i	2.8	11.0	19.5	28.0
	ii	1.4	5.5	9.7	14.0
	iii	0.7	2.8	4.9	7.0
1–10	i	283.4	97.8	151.0	204.1
	ii	141.7	48.9	75.5	102.1
	iii	70.9	24.5	37.7	51.0
10–20	i	1070.7	157.1	341.9	526.7
	ii	535.3	78.6	171.0	263.4
	iii	267.7	39.3	85.5	131.7
20–50	i	238.0	233.0	294.7	356.5
	ii	119.0	116.5	147.4	178.2
	iii	59.5	58.3	73.7	89.1
50–100	i	1668.0	449.9	557.4	664.9
	ii	834.0	225.0	278.7	332.4
	iii	417.0	112.5	139.3	166.2
>100	i	4312.7	950.0	1409.7	1869.3
	ii	2156.3	475.0	704.8	934.7
	iii	1078.2	237.5	352.4	467.3
**Total**	**i**	**7575.5**	**1898.8**	**2774.2**	**3649.5**
	**ii**	**3787.8**	**949.4**	**1387.1**	**1824.8**
	**iii**	**1893.9**	**474.7**	**693.5**	**912.4**

**Table 4:** Statistics and forecasts of the power generation capacity registered in wind projects.

Power range (MW)	Approval process stage	PGC registered in projects (MW)				PGC forecasted in future projects (MW)			
		2016	2017	2018	2019	2020	2021	2022	2023
0–10	1	9.9	9.9	11.9	9.9	17.3	24.6	32.0	39.3
	2	0.0	0.0	4.8	19.8	9.9	17.3	24.6	32.0
	3	0.0	0.0	0.0	0.0	19.8	9.9	17.3	24.6
10–20	1	0.0	19.9	27.9	0.0	8.5	16.9	25.4	33.8
	2	39.8	19.9	9.6	0.0	0.0	8.5	16.9	25.4
	3	19.9	39.8	59.7	0.0	0.0	0.0	8.5	16.9
20–100	1	0.0	0.0	0.0	225.0	264.8	304.6	344.3	384.1
	2	0.0	0.0	0.0	753.8	225.0	264.8	304.6	344.3
	3	0.0	0.0	0.0	0.0	753.8	225.0	264.8	304.6
>100	1	344.0	412.0	453.2	703.0	1041.1	1379.2	1717.3	2055.4
	2	0.0	0.0	197.8	1041.2	703.0	1041.1	1379.2	1717.3
	3	0.0	0.0	0.0	0.0	1041.2	703.0	1041.1	1379.2
**Total**	**1**	**353.9**	**441.8**	**493.0**	**937.9**	**1331.6**	**1725.3**	**2119.0**	**2512.7**
	**2**	**39.8**	**19.9**	**212.2**	**1814.8**	**937.9**	**1331.6**	**1725.3**	**2119.0**
	**3**	**19.9**	**39.8**	**59.7**	**0.0**	**1814.8**	**937.9**	**1331.6**	**1725.3**

**Table 5:** Scenarios of power generation capacities yearly integrated into the electric system. Wind projects.

Power range (MW)	Scenario	Forecasted implementation of PGC (MW)			
		2020	2021	2022	2023
0–10	i	19.8	9.9	17.3	24.6
	ii	9.9	5.0	8.6	12.3
	iii	5.0	2.5	4.3	6.2
10–20	i	0.0	0.0	8.5	16.9
	ii	0.0	0.0	4.2	8.5
	iii	0.0	0.0	2.1	4.2
20–100	i	753.8	225.0	264.8	304.6
	ii	376.9	112.5	132.4	152.3
	iii	188.5	56.3	66.2	76.1
>100	i	1041.2	703.0	1041.1	1379.2
	ii	520.6	351.5	520.6	689.6
	iii	260.3	175.8	260.3	344.8
**Total**	**i**	**1814.8**	**937.9**	**1331.6**	**1725.3**
	**ii**	**907.4**	**469.0**	**665.8**	**862.6**
	**iii**	**453.7**	**234.5**	**332.9**	**431.3**

**Table 6:** Statistics and forecasts of the power generation capacity registered in biomass projects.

Power range (MW)	Approval process stage	PGC registered in projects (MW)				PGC forecasted in future projects (MW)			
		2016	2017	2018	2019	2020	2021	2022	2023
0–1	1	1.7	2.6	4.0	0.5	4.2	7.9	11.6	15.3
	2	0.0	0.0	1.3	0.0	0.5	4.2	7.9	11.6
	3	0.0	0.0	0.0	0.0	0.0	0.5	4.2	7.9
1–10	1	10.9	14.6	21.9	0.0	19.8	39.5	59.3	79.1
	2	0.0	20.4	7.0	28.8	0.0	19.8	39.5	59.3
	3	0.0	0.0	20.4	0.0	28.8	0.0	19.8	39.5
10–20	1	0.0	0.0	0.0	0.0	0.0	0.0	0.0	0.0
	2	0.0	0.0	0.0	19.8	0.0	0.0	0.0	0.0
	3	0.0	0.0	0.0	0.0	19.8	0.0	0.0	0.0
20–50	1	25.0	25.0	37.5	0.0	36.5	73.0	109.5	146.0
	2	20.6	45.4	12.0	25.0	0.0	36.5	73.0	109.5
	3	0.0	41.6	87.0	0.0	25.0	0.0	36.5	73.0
>50	1	0.0	0.0	0.0	0.0	0.0	0.0	0.0	0.0
	2	0.0	0.0	0.0	0.0	0.0	0.0	0.0	0.0
	3	0.0	0.0	0.0	0.0	0.0	0.0	0.0	0.0
**Total**	**1**	**37.6**	**42.2**	**63.4**	**0.5**	**60.5**	**120.5**	**180.4**	**240.4**
	**2**	**20.6**	**65.8**	**20.3**	**73.5**	**0.5**	**60.5**	**120.5**	**180.4**
	**3**	**0.0**	**41.6**	**107.4**	**0.0**	**73.5**	**0.5**	**60.5**	**120.5**

**Table 7:** Scenarios of power generation capacities yearly integrated into the electric system. Biomass projects.

Power range (MW)	Scenario	Forecasted implementation of PGC (MW)				
		2019	2020	2021	2022	2023
0–1	i	0.0	0.5	4.2	7.9	0.0
	ii	0.0	0.3	2.1	3.9	0.0
	iii	0.0	0.1	1.1	2.0	0.0
1–10	i	28.8	0.0	19.8	39.5	28.8
	ii	14.4	0.0	9.9	19.8	14.4
	iii	7.2	0.0	4.9	9.9	7.2
10–20	i	19.8	0.0	0.0	0.0	19.8
	ii	9.9	0.0	0.0	0.0	9.9
	iii	4.9	0.0	0.0	0.0	4.9
20–50	i	25.0	0.0	36.5	73.0	25.0
	ii	12.5	0.0	18.3	36.5	12.5
	iii	6.3	0.0	9.1	18.3	6.3
>50	i	0.0	0.0	0.0	0.0	0.0
	ii	0.0	0.0	0.0	0.0	0.0
	iii	0.0	0.0	0.0	0.0	0.0
**Total**	**i**	**73.5**	**0.5**	**60.5**	**120.5**	**73.5**
	**ii**	**36.8**	**0.3**	**30.2**	**60.2**	**36.8**
	**iii**	**18.4**	**0.1**	**15.1**	**30.1**	**18.4**

**Table 8:** Statistics and forecasts of the power generation capacity registered in SHC projects.

Power range (MW)	Approval process stage	PGC registered in projects (MW)				PGC forecasted in future projects (MW)			
		2016	2017	2018	2019	2020	2021	2022	2023
0–1	1	1.1	1.1	1.7	1.0	3.1	5.1	7.2	9.2
	2	0.0	0.0	0.5	0.8	1.0	3.1	5.1	7.2
	3	0.0	0.0	0.0	1.0	0.8	1.0	3.1	5.1
1–10	1	109.4	214.7	386.5	73.3	402.9	732.5	1062.1	1391.7
	2	66.7	109.4	103.1	19.8	73.3	402.9	732.5	1062.1
	3	15.1	70.2	179.6	1.0	19.8	73.3	402.9	732.5
10–20	1	0.0	398.0	557.2	112.7	561.7	1010.6	1459.6	1908.6
	2	194.0	0.0	191.0	185.9	112.7	561.7	1010.6	1459.6
	3	0.0	194.0	194.0	0.0	185.9	112.7	561.7	1010.6
**Total**	**1**	**110.5**	**613.8**	**945.4**	**187.0**	**967.6**	**1748.2**	**2528.9**	**3309.5**
	**2**	**260.7**	**109.4**	**294.6**	**206.5**	**187.0**	**967.6**	**1748.2**	**2528.9**
	**3**	**15.1**	**264.2**	**373.6**	**2.0**	**206.5**	**187.0**	**967.6**	**1748.2**

**Table 9:** Scenarios of power generation capacities yearly integrated into the electric system. SHC projects.

Power range (MW)	Scenario	Forecasted implementation of PGC (MW)				
		2019	2020	2021	2022	2023
0–1	i	0.8	1.0	3.1	5.1	0.8
	ii	0.4	0.5	1.5	2.6	0.4
	iii	0.2	0.3	0.8	1.3	0.2
1–10	i	19.8	73.3	402.9	732.5	19.8
	ii	9.9	36.7	201.4	366.2	9.9
	iii	5.0	18.3	100.7	183.1	5.0
10–20	i	185.9	112.7	561.7	1010.6	185.9
	ii	93.0	56.3	280.8	505.3	93.0
	iii	46.5	28.2	140.4	252.7	46.5
**Total**	**i**	**206.5**	**187.0**	**967.6**	**1748.2**	**206.5**
	**ii**	**103.3**	**93.5**	**483.8**	**874.1**	**103.3**
	**iii**	**51.6**	**46.7**	**241.9**	**437.1**	**51.6**

**Table 10:** Scenarios of non-conventional power generation capacities yearly integrated into the electric system.

Power range (MW)	Scenario	Forecasted PGC (MW)				
		2019	2020	2021	2022	2023
0–1	i	3.6	12.6	26.8	41.0	3.6
	ii	1.8	6.3	13.4	20.5	1.8
	iii	0.9	3.1	6.7	10.2	0.9
1–10	i	351.8	181.0	590.9	1000.8	351.8
	ii	175.9	90.5	295.4	500.4	175.9
	iii	87.9	45.3	147.7	250.2	87.9
10–20	i	1276.3	269.8	912.0	1554.3	1276.3
	ii	638.2	134.9	456.0	777.1	638.2
	iii	319.1	67.4	228.0	388.6	319.1
20–50	i	263.0	233.0	331.2	429.5	263.0
	ii	131.5	116.5	165.6	214.7	131.5
	iii	65.8	58.3	82.8	107.4	65.8
50–100	i	2421.8	674.9	822.2	969.4	2421.8
	ii	1210.9	337.5	411.1	484.7	1210.9
	iii	605.5	168.7	205.5	242.4	605.5
>100	i	5353.9	1653.0	2450.8	3248.5	5353.9
	ii	2676.9	826.5	1225.4	1624.3	2676.9
	iii	1338.5	413.3	612.7	812.1	1338.5
**Total**	**i**	**9670.4**	**3024.2**	**5133.9**	**7243.5**	**9670.4**
	**ii**	**4835.2**	**1512.1**	**2566.9**	**3621.7**	**4835.2**
	**iii**	**2417.6**	**756.1**	**1283.5**	**1810.9**	**2417.6**

## 2 Experimental design, materials, and methods

The PGC of the NCRE projects between 2016 and 2019 shown in Table 1 is used to interpolate the performance of the generation capacity registered in NCRE projects in Colombia. The interpolation is used to forecast the PGC in NCRE projects to be registered between 2020 and 2023. Projects in the NCRE database goes through the three approval stages defined by the government [3].

• Preliminary feasibility assessment: a preliminary study to develop the environmental impact assessment, and the technical and economic feasibility of the project (it takes around two years).
• Complete feasibility assessment: assessment of the technical, economic, environmental and social feasibility of the project (it takes stage takes up to one year).
• Pre-implementation: completion of the final design of the project, and definition of the implementation schedule. The project changes to the status “*ready for implementation*” (it takes up to one year).

In total, it takes about four years between the registration of a project to the database and the clearance of the government for its implementation [3]. In addition, it takes around one year to implement the project after its approval [4,5]. Thus, it takes around 5 years from registering a project to its implementation. This average time is used to forecast the initial exploitation date of the projects registered between 2016 and 2019. The PGC yearly accumulated for each NCRE source is forecasted by adding the generation capacity of the projects after five years, considering the different scenarios of project success.

Overall, between 70 and 75% of the renewable-based power generation projects registered at UPME are approved for implementation [6]. Thus, three scenarios considering a high (100%), medium (50%) and low (25%) implementation of these projects were considered:

i. Scenario 1 (high success): 100% of the power generation capacities of NCRE projects registered are implemented.
ii. Scenario 2 (medium success): 50% of the power generation capacities of NCRE projects registered are implemented.
iii. Scenario 3 (low success): 25% of the power generation capacities of NCRE projects registered are implemented.

## Conflict of Interest

The authors declare that they have no known competing financial interests or personal relationships that could have appeared to influence the work reported in this paper.
